# Dimensional Accuracy and Surface Quality of AZ91D Magnesium Alloy Components after Precision Milling

**DOI:** 10.3390/ma14216446

**Published:** 2021-10-27

**Authors:** Jarosław Korpysa, Józef Kuczmaszewski, Ireneusz Zagórski

**Affiliations:** Department of Production Engineering, Faculty of Mechanical Engineering, Lublin University of Technology, 20-618 Lublin, Poland; j.kuczmaszewski@pollub.pl (J.K.); i.zagorski@pollub.pl (I.Z.)

**Keywords:** magnesium alloys, precision milling, dimensional accuracy, surface roughness

## Abstract

This study investigates a precision milling process conducted with the use of conventional end mills and a standard CNC (Computer Numerical Control) machine tool. Milling tests were performed on samples of AZ91D magnesium alloy using TiB_2_- and TiAlN-coated three-edge end mills measuring 16 mm in diameter. The following technological parameters were made variable: cutting speed, feed per tooth and axial depth of cut. The effects of precision milling were evaluated by analysing the scatter of dimension values obtained in successive tool passes. In addition to that, deviations from the assumed nominal depth as well as obtained ranges of dimension varation were analysed. The study also examined surface quality obtained in the precision milling process, based on the basic surface roughness parameters: Ra, Rz and RSm. Results have confirmed that the use of conventional cutting tools and a standard CNC machine tool makes it possible to manufacture components characterized by relatively small scatter of dimension values and high accuracy classes. Additionally, the results have shown that the type of tool coating and variations of individual technological parameters exert impact on the dimensional accuracy and surface quality obtained.

## 1. Introduction

Magnesium alloys are characterized by a low density of only 1.74 g/cm^3^, which is one of the reasons for a continuous increase in the use of these construction materials. These alloys are predominantly used in the aviation and automotive industries where reduced element weight is of key importance. This mainly results from the need to reduce fuel consumption. Owing to their capacity for damping electromagnetic waves, magnesium alloys are also used in the electronics industry. In addition, the biocompatibility and biodegradability properties of these materials render them valuable for the biomedical sector. The advantages of magnesium alloys also include good mechanical properties, high vibration damping capability and resistance to atmospheric corrosion [1,2,3]. Studies on the milling processes for magnesium alloys confirm that these materials have very good machinability and that it is possible to obtain high quality surface comparable to that obtained by abrasive machining [4,5].

Requirements set by the market for manufactured elements are more and more demanding, which is entails continuous improvement of machining processes in order to obtain surfaces with higher and higher manufacturing accuracy and quality. As far as components made of magnesium alloys are concerned, the use of abrasive machining for improving manufacturing quality is quite limited, primarily due to the occurrence of magnesium dust that is formed during this type of machining. Magnesium dust poses a high risk both to the health of machine operators and to the condition of machines themselves; additionally, it has explosive properties, which is why it is necessary to obtain the highest possible accuracy of components via milling [2,6]. An alternative solution seems to be the use of precision milling.

In terms of accuracy, milling is generally divided into conventional, precision and ultra-precision. Although this division mainly rests on the undeformed chip thickness, it can also be based on the dimensional accuracy of manufactured elements, their surface topography or the size of cutting tools used [7,8]. Precision milling is conducted with relatively low technological parameters such as the depth of cut and the feed per tooth, their values being comparable to the cutting edge radius r_n_ [7,8,9]. Unlike in conventional milling where the cutting edge is treated as completely sharp, the cutting edge radius r_n_ is an important factor in precision milling. The use of an undeformed chip thickness value that is too low in relation to the cutting edge radius r_n_ causes that the material does not undergo cutting but merely ploughing. This phenomenon is highly undesired due to considerable deterioration in workpiece surface condition, which is manifested by the presence of characteristic mill marks [10,11]. Many studies investigate phenomena occurring during the precision milling process conducted with different f_z_/r_n_ ratios, which makes it possible to examine the transition between ploughing and material cutting [10,12,13,14,15,16].

The use of precision milling enables the production of components with high surface quality, and thus eliminates the need for additional finishing operations [17]. Studies [11,18,19,20,21,22,23] conducted on aluminium and titanium alloys show that their surface condition predominantly depends on the precision milling parameters, the most important being feed per tooth as it determines the initiation of the cutting process. A too low feed per tooth value causes ploughing and, in effect, a significant deterioration in the workpiece surface condition. The lowest surface roughness parameters are obtained in the transition zone, while further increase in feed per tooth leads to increased surface roughness. Improved surface quality is usually achieved by increasing the cutting speed, while the effect of the depth of cut on the surface quality usually depends on other milling conditions. Apart from technological parameters, the type of cutting tools is another factor that has the main impact on the obtained surface quality. Precision milling processes for different engineering materials are often conducted using tools made of cemented carbides [11,12,20,21,22,23,24] that are also very effective in conventional milling of magnesium alloys [25]. These tools are much cheaper than diamond cutters, and the use of fine-grained carbides enables the production of precise tools with equally low values of the cutting edge radius r_n_. The cutting ability of tools can be additionally improved by the application of special protective tool coatings, and their appropriate selection has impact on the effects of machining [26,27,28]. Other factors affecting workpiece surface condition include: cooling method [10,29], milling direction [16,20], tool length [30] and cutting tool inclination angle [31,32].

A review of the literature on precision milling shows that steels are the most frequently investigated material in general, while aluminium and titanium alloys are the most often investigated light metal alloys. What is more, most studies are conducted with the use of micro tools and mainly focused on examining surface condition. The novelty of this study is that it investigates whether precision milling can be conducted using conventional tools and a standard CNC machine tool. Moreover, the study investigates the problem of achievable dimensional accuracy of manufactured elements, so far lacking in the scientific literature. In addition to that, the material tested in this study is magnesium alloy, and—to our knowledge—no previous study has investigated the precision milling process for this material. The possibility of using conventional tools in precision milling will increase the efficiency of such processes, when compared to the processes performed with the use of micro tools. The study will also contribute to the development of an optimal combination of milling parameters, as well as to the establishment of a relationship between the tool coating type applied and the milling effects obtained.

## 2. Materials and Methods

**Figure 1:**
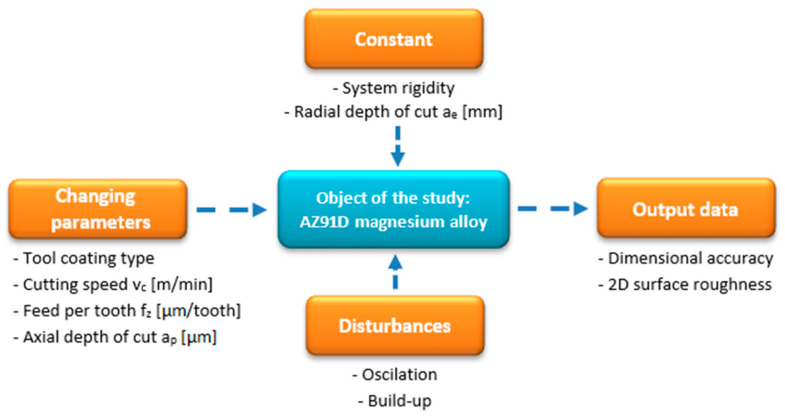
Research plan.

The primary objective of the study conducted in compliance with the research plan shown in Figure 1 was to determine the effects of tool coating type and technological parameters on the surface quality and dimensional accuracy of manufactured components.

**Figure 2:**
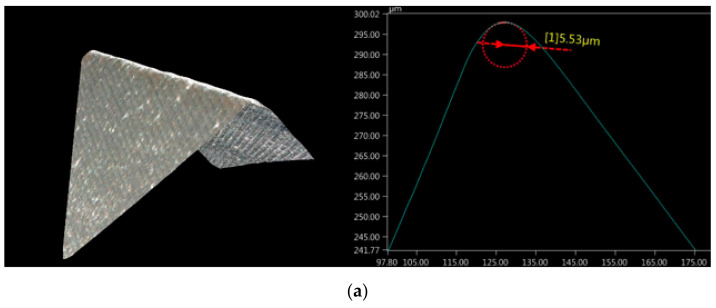

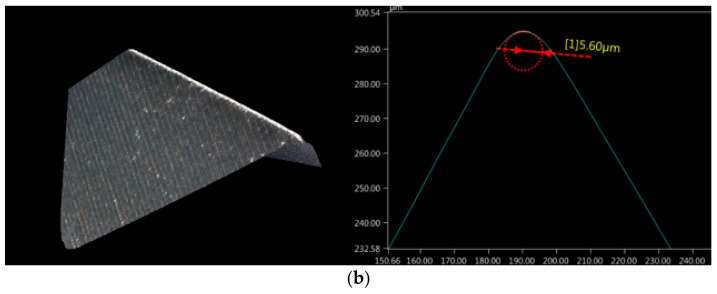
Cutting edge and its radius r_n_ in a tool coated with (**a**) TiB_2_ and (**b**) TiAlN.

The milling process for AZ91D magnesium alloy samples was performed using the VMC 800 HS vertical milling centre from AVIA (Warsaw, Poland). This machine tool is equipped with an electrospindle having a maximum speed of 24,000 rpm, which makes it possible to perform HSC machining for most technological cases. Tests were performed using two conventional tools with a diameter of 16 mm, suitable for wide industrial use, i.e., AM3SSD1600A100 end mills from Mitsubishi (Tokyo, Japan), which are suited for machining of light metal alloys. The tools are made of ultra-micrograin carbide, which ensures their high manufacturing accuracy. The tools were coated with TiB_2_ and TiAlN; such tool coatings are widely used in the machining of magnesium alloys. The tools have three cutting edges to ensure continuous cutting. Prior to the milling tests, the cutting edge r_n_ of both tools was measured (Figure 2). The mean values calculated from 21 measurements made at equal, several-micrometre intervals along the cutting edge were as follows: r_n_ = 5.53 µm for the TiB_2_-coated tool and r_n_ = 5.60 µm for the TiAlN-coated tool. The measurements were conducted with the use of the Keyence VHX-5000 microscope (Osaka, Japan) at a thousand-fold magnification.

The tools were mounted in the spindle using Celsio heat shrinking toolholders from Schunk (Lauffen, Germany) provided with HSK-A63 interface. The toolholding system was balanced in the quality grade G2.5 at 25,000 rpm according to ISO 21940-11:2016, using the Cimat RT 610 balancer (Bydgoszcz, Poland). Selected technological parameters were made variable in the tests. The cutting speed was varied in the range v_c_ = 400–1200 m/min, the feed per tooth f_z_ ranged 1–9 µm/tooth, the axial depth of cut a_p_ ranged 60–100 µm, while the radial depth of cut a_e_ was maintained constant at 14 mm. The range of feed per tooth f_z_ was chosen to include both lower and greater values than the value of the cutting edge radius r_n_, whereas the range of cutting speeds v_c_ was limited by the maximum spindle speed of the machine tool, which is 24,000 rpm. To compare results of precision and conventional milling, the tests were conducted using the following technological parameters: precision milling—v_c_ = 400 m/min, f_z_ = 5 µm/tooth, a_p_ = 100 µm; and conventional milling—v_c_ = 400 m/min, f_z_ = 50 µm/tooth, a_p_ = 1000 µm. The machining process was carried out as dry milling, without cooling or lubrication.

**Figure 3:**
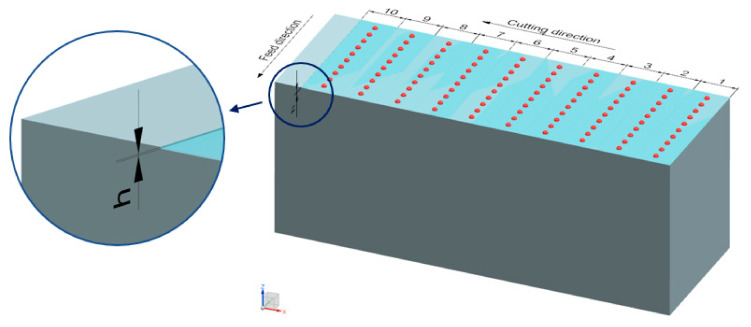
Scheme of the analysed milling process and dimensional accuracy measurement.

Dimensional accuracy measurements were conducted on the machining centre immediately after the milling process in order to avoid errors related to workpiece mounting. Measurements were repeated ten times per tool pass, which made it possible to determine the scatter and repeatability of dimensional accuracy results. Measurements were made along the Z axis of the machine tool according to the scheme shown in Figure 3, using the Heidenhain TS 640 workpiece touch probe (Traunreut, Germany) making part of the milling centre.

Measurements of the basic surface roughness parameters were made using the Hommel Tester T1000 contact profilometer by Jenoptik (Jena, Germany) and a Gauss filter (M1), in compliance with the ISO 16610-21:2013-02 standard. The sampling length lr was set to 0.8 mm, the measuring length ln was 4 mm, and the traverse speed v_t_ was equal to 0.5 mm/s. The measurements were repeated five times for every tool pass, which made it possible to estimate mean values and standard deviation of the analysed surface roughness parameters.

## 3. Results and Discussion

### 3.1. Dimensional Accuracy

**Figure 4:**
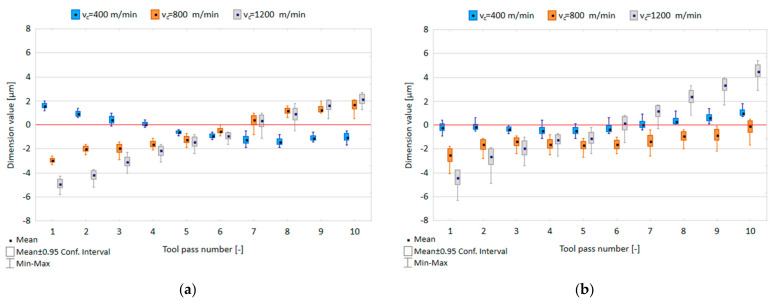
Cutting speed v_c_ versus dimensional accuracy in the milling process performed using a tool coated with: (**a**) TiB_2_ and (**b**) TiAlN; (f_z_ = 5 µm/tooth; a_p_ = 80 µm).

The effect of the cutting speed v_c_ and tool coating type on the dimensional accuracy obtained in precision milling is shown in Figure 4.

In Figure 4, the red line marks the nominal dimension after the final finishing tool pass at a_p_ equal to 0.08 mm. It can be observed that for both tools, the scatter of this dimension values as measured for individual tool passes increases with increasing the cutting speed, ranging 0.5–1.2 µm, 0.7–1.6 µm and 1.0–2.3 µm for the TiB_2_-coated tool, and 0.7–1.5 µm, 1.4–2.3 µm and 1.9–3.0 µm for the TiAlN-coated tool. Despite the relatively small scatter of results, which does not exceed 3.75% of the assumed nominal depth of 80 µm, the smallest deviation was obtained for both tools when the milling process was conducted with the lowest tested cutting speed of v_c_ = 400 m/min. An increase in the cutting speed caused a clear change in the dimensional values measured for successive tool passes relative to the nominal depth value. The range of dimension variation of the entire surface increased with increasing the cutting speed from 3.9 µm to 8.5 µm for the TiB_2_-coated tool and from 2.9 µm to 11.7 µm for the TiAlN-coated tool.

**Figure 5:**
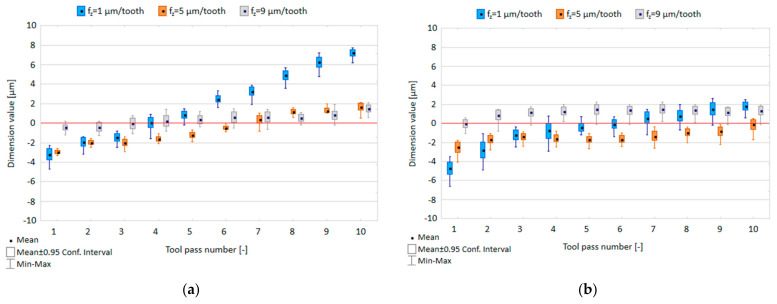
Feed per tooth f_z_ versus dimensional accuracy in the milling process conducted using a tool coated with: (**a**) TiB_2_ and (**b**) TiAlN; (v_c_ = 800 m/min; a_p_ = 80 µm).

The effect of the feed per tooth f_z_ and tool coating type on the dimensional accuracy obtained in precision milling is shown in Figure 5.

Both the highest scatter of dimension values obtained in successive tool passes ranging 1.5–2.5 µm for the TiB_2_-coated tool and 1.9–3.8 µm for the TiAlN-coated tool, as well as the largest deviation from the nominal depth were obtained when the milling process was conducted with the lowest feed per tooth value of f_z_ = 1 µm/tooth. An increase in the feed per tooth led reduced the scatter of the dimension values to 0.7–1.8 µm and 1.3–2.2 µm for the milling process performed with the TiB_2_ -coated tool and to 1.4–2.3 µm and 1.7–2.3 µm for the TiAlN-coated tool. Hence, the scatter of the obtained dimension values does not exceed 4.75% of the assumed nominal depth of 80 µm, while the deviation of the measured dimensions from the nominal depth can be observed for successive tool passes. Considering the entire surface, due to increased feed per tooth, the range of dimension variation decreased from 12.4 µm to 3.4 µm (TiB_2_-coated tool) and from 9.2 µm to 3.3 µm (TiAlN-coated tool). Increased feed per tooth has a stabilizing effect on the dimension variability range, which may be due to the milling process dynamics (higher spindle speed).

**Figure 6:**
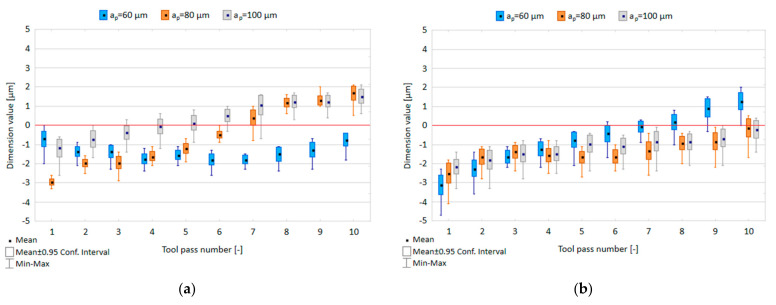
Axial depth of cut a_p_ versus dimensional accuracy in the milling process conducted using a tool coated with: (**a**) TiB_2_ and (**b**) TiAlN; (v_c_ = 800 m/min; f_z_ = 5 µm/tooth).

The effect of the axial depth of cut a_p_ and tool coating type on the dimensional accuracy in precision milling is shown in Figure 6.

Regardless of the depth of cut applied, the scatter of the dimension values obtained in successive tool passes is at a similar level, ranging 0.8–2.0 µm, 0.7–1.8 µm and 1.3–2.3 µm for the TiB_2_-coated tool and 1.1–2.4 µm, 1.4–2.3 µm and 1.7–2.3 µm for the TiAlN-coated tool. Due to a change in the axial depth of cut at which the milling process was performed, the scatter of results obtained for subsequent depths of cut does not, however, exceed 4%, 2.9% and 2.3% of the nominal depth value. Nevertheless, the plots show a clear increase in the dimension values for successive tool passes, which causes a gradual deviation from the assumed nominal depth. The range of the dimension variation obtained for the entire surface with increased axial depth of cut was within 2.6–5.4 µm for the TiB_2_-coated tool, but they show a decrease from 6.7 µm to 3.7 µm for the TiAlN-coated tool.

**Figure 7:**
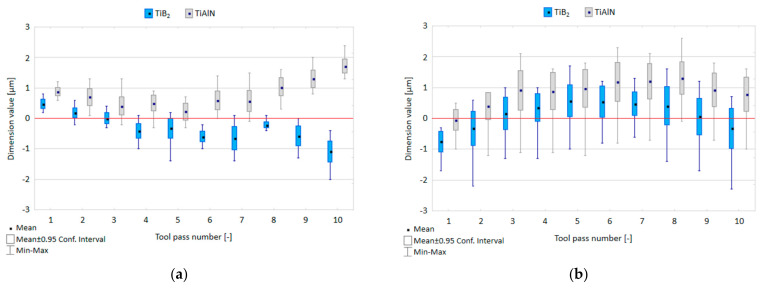
Dimensional accuracy obtained in: (**a**) precision milling, (**b**) conventional milling performed using the tools with TiB_2_ and TiAlN coatings.

Figure 7 shows the relationship between tool coating type and dimensional accuracy in precision and conventional milling processes.

In the precision milling process, the scatter of dimension values ranges 0.5–1.6 µm for the TiB_2_-coated tool and 0.6–1.6 µm for the TiAlN-coated tool; therefore, it does not exceed 1.6% of the assumed nominal depth of 100 µm. It can be observed that in successive tool passes the dimension values gradually decrease when the TiB_2_-coated tool is used; however, the application of the TiAlN-coated tool leads to an increase in the dimension values. The range of the dimension variation obtained for the entire surface is 2.8 µm and 2.7 µm for the TiB_2_-coated tool and the TiAlN-coated tool, respectively. In the conventional milling process, the scatters of dimension values are similar for both tools and range within 1.4–3 µm and 1.5–3.1 µm for the TiB_2_-coated tool and the TiAlN-coated tool, respectively. With successive tool passes, the dimension values oscillate near the assumed nominal depth of 1 mm, reaching the dimension variability range of 4 µm for the TiB_2_-coated tool and 3.8 µm for the TiAlN-coated tool.

An analysis of the results of dimensional accuracy studies has shown the main influence of two technological parameters: cutting speed and feed per tooth. An appropriate change of these two parameters makes it possible to reduce the scatter of the dimension values for individual tool passes, as well as to reduce the dimension variability range analysed with respect to the entire machined surface. The change in axial depth of cut, as well as the type of cutting tool, did not have a pronounced impact on the machining effect.

**Figure 8:**
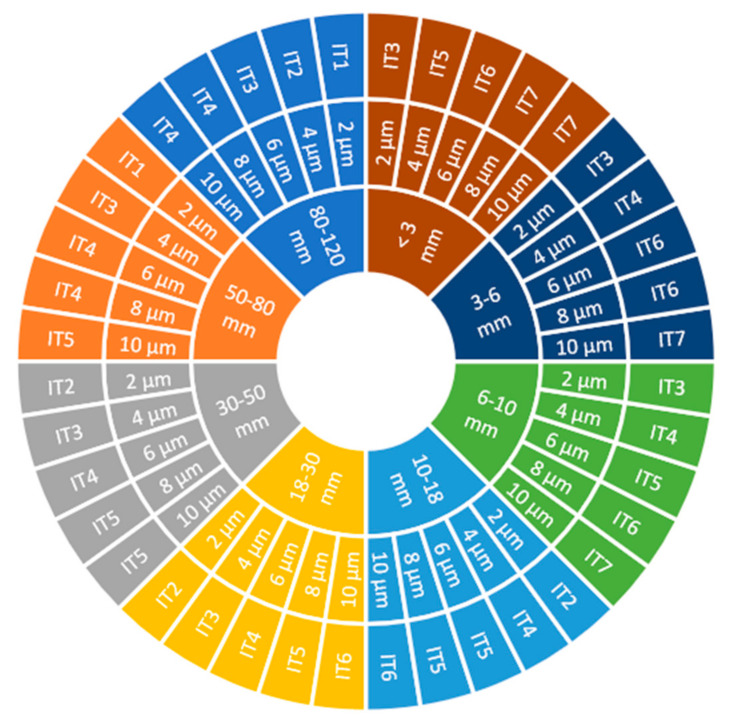
Standard tolerance classes IT for the tolerance of 2 µm, 4 µm, 6 µm, 8 µm and 10 µm, depending on the nominal dimension ranging 1–120 mm.

Under ISO 286-2:2010, the achievable standard tolerance class is defined by the nominal dimension of the workpiece and the tolerance range for this dimension (Figure 8).

A comparison of the dimensional accuracy results obtained for a sample height of approx. 100 mm reveals that, depending on the technological parameters, the tolerance classes IT2–IT5 were achieved for both cutting tools used. Along with increasing the workpiece size, the tolerance range increases too, and thus—despite the same scatter of dimensional accuracy results—the achieved tolerance class is higher.

### 3.2. Surface Roughness

**Figure 9:**
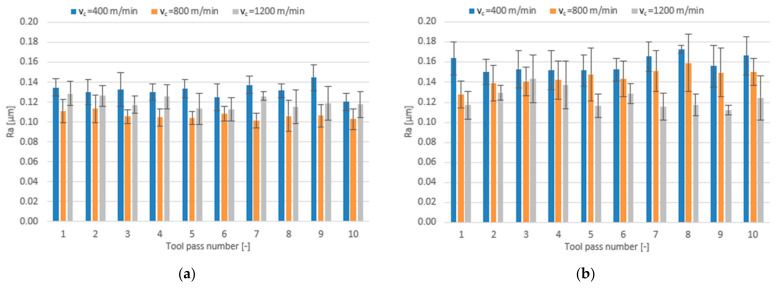
Cutting speed v_c_ versus surface roughness parameter Ra in the milling process performed using a tool coated with: (**a**) TiB_2_ and (**b**) TiAlN; (f_z_ = 5 µm/tooth; a_p_ = 80 µm).

**Figure 10:**
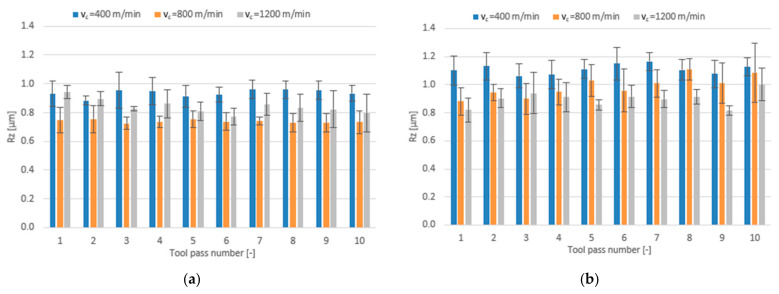
Cutting speed v_c_ versus surface roughness parameter Rz in the milling process performed using a tool coated with: (**a**) TiB_2_ and (**b**) TiAlN; (f_z_ = 5 µm/tooth; a_p_ = 80 µm).

**Figure 11:**
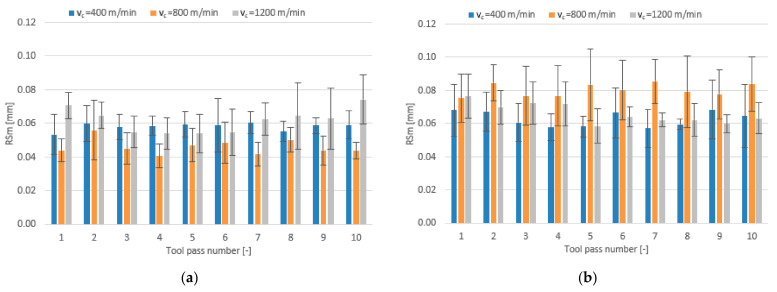
Cutting speed v_c_ versus surface roughness parameter RSm in the milling process performed using a tool coated with: (**a**) TiB_2_ and (**b**) TiAlN; (f_z_ = 5 µm/tooth; a_p_ = 80 µm).

In addition to investigating the effects of individual technological parameters and tool coating type on the dimensional accuracy results, the influence of these factors on the surface roughness parameters Ra, Rz and RSm was examined, too. Figure 9, Figure 10 and Figure 11 show the bar charts illustrating the impact of the cutting speed v_c_ and tool coating type on the analysed surface roughness parameters.

The mean values of the Ra parameter obtained in the milling process performed with the TiB_2_-coated tool first decrease with increasing the cutting speed to v_c_ = 800 m/min and then increase with a further increase in the cutting speed. For the surfaces milled with the TiAlN-coated tool, the mean values of Ra decrease over the entire range of the cutting speed; nevertheless, they are higher than the values obtained for the surfaces milled with the TiB_2_-coated tool and are additionally characterized by a greater scatter of results. Regardless of the cutting speed used, the mean values of the Ra parameter are within very narrow ranges of 0.106–0.144 µm for the tool with a TiB_2_ coating and 0.113–0.173 µm for the tool with a TiAlN coating.

Increasing the cutting speed to v_c_ = 800 m/min in the milling process performed with the TiB_2_-coated tool results in a decrease in the mean value of the surface roughness parameter Rz, while a further increase in the cutting speed leads to an increase in the mean value of Rz. When the milling process is performed with the TiAlN-coated tool, the mean values of Rz decrease over the entire cutting speed range. Nevertheless, the mean values of Rz obtained for this tool are, in most cases, higher (0.816–1.166 µm) than those obtained for the TiB_2_-coated tool (0.724–0.958 µm).

For the milling process performed using the TiB_2_-coated tool, one can observe a decrease in the mean value of the surface roughness parameter RSm at the intermediate cutting speed v_c_ = 800 m/min and an increase in this parameter at the highest cutting speed. An opposite phenomenon can be observed for the milling process performed using the TiAlN-coated tool, because the highest values of the RSm parameter are obtained at the cutting speed set to v_c_ = 800 m/min. The mean values of RSm range within 0.041–0.074 mm for the TiB_2_-coated tool and 0.041–0.074 mm for the TiAlN-coated tool. The values obtained with both tools for the milling process conducted with the lowest and highest cutting speeds are very similar; however, due to the RSm decrease occurring at the intermediate cutting speed value, a more favourable surface roughness is obtained with the tool with a TiB_2_ coating.

**Figure 12:**
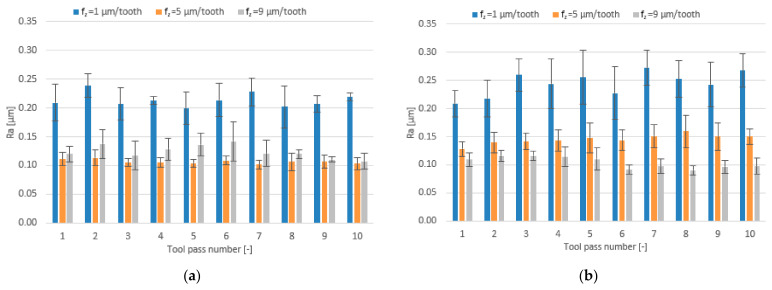
Feed per tooth f_z_ versus surface roughness parameter Ra in the milling process performed using a tool coated with: (**a**) TiB_2_ and (**b**) TiAlN; (v_c_ = 800 m/min; a_p_ = 80 µm).

**Figure 13:**
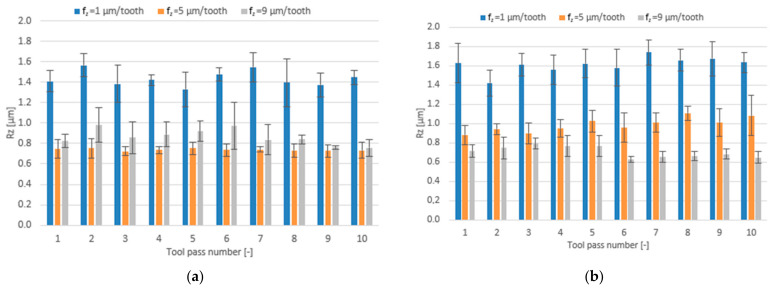
Feed per tooth f_z_ versus surface roughness parameter Rz in the milling process performed using a tool coated with: (**a**) TiB_2_ and (**b**) TiAlN; (v_c_ = 800 m/min; a_p_ = 80 µm).

**Figure 14:**
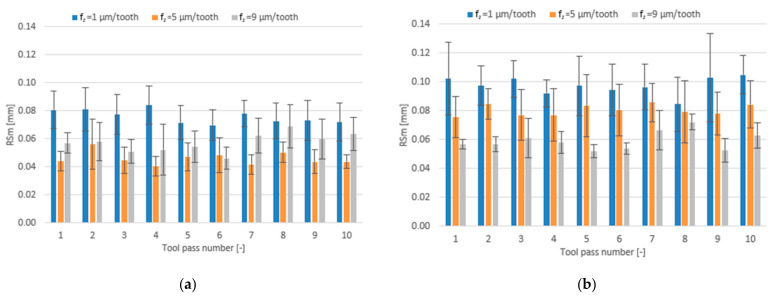
Feed per tooth f_z_ versus surface roughness parameter RSm in the milling process performed using a tool coated with: (**a**) TiB_2_ and (**b**) TiAlN; (v_c_ = 800 m/min; a_p_ = 80 µm).

The effect of the feed per tooth range f_z_ = 1–9 µm/tooth and different tool coating types on surface roughness parameters is presented in the form of bar charts in Figure 12, Figure 13 and Figure 14.

Regardless of the tool coating used, the highest mean values of the Ra parameter are obtained when the milling process is conducted with the lowest feed per tooth of f_z_ = 1 µm/tooth. For the milling process performed with the TiB_2_-coated tool, an increase in the feed per tooth results first in a decrease in the mean value of the roughness parameter Ra and then in an increase but to a smaller extent. The mean values of Ra obtained for this tool range from 0.101 to 0.238 µm. For the tool with a TiAlN coating, the mean values of the Ra parameter decrease over the entire tested range of feed per tooth and amount to 0.090–0.272 µm.

Similar phenomena can be observed for the surface roughness parameter Rz. The mean values of Rz obtained in the milling process performed with the TiB_2_-coated tool initially decrease with increasing the feed per tooth and then increase. On the other hand, when the milling process is performed with the TiAlN-coated tool, the mean values of the Rz parameter decrease over the entire tested feed per tooth range. The mean values of Rz range within 0.73–1.57 µm for the TiB_2_ coating and 0.63–1.74 µm for the TiAlN coating.

A relationship between the feed per tooth and the roughness parameter RSm is similar to that established for the Ra and Rz parameters. On increasing the feed per tooth when milling with the TiB_2_-coated tool, the mean values of the RSm parameter decrease first and then increase. The mean values of the RSm parameter obtained for this tool range from 0.041 mm to 0.084 mm, while for the tool with a TiAlN coating they range from 0.052 mm to 0.105 mm, gradually decreasing with increasing the feed per tooth.

**Figure 15:**
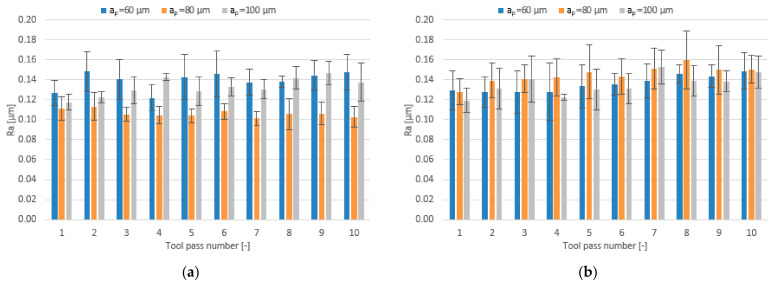
Axial depth of cut a_p_ versus surface roughness parameter Ra in the milling process performed using a tool coated with: (**a**) TiB_2_ and (**b**) TiAlN; (v_c_ = 800 m/min; f_z_ = 5 µm/tooth).

**Figure 16:**
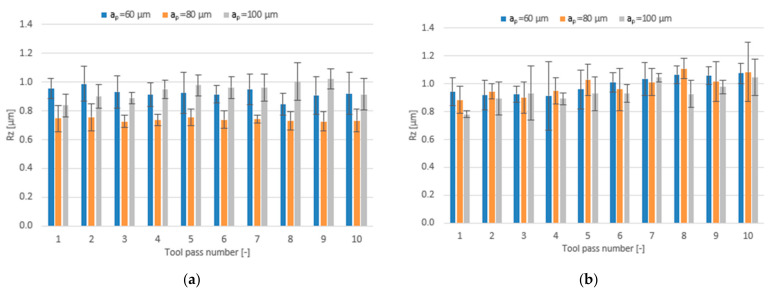
Axial depth of cut a_p_ versus surface roughness parameter Rz in the milling process performed using a tool coated with: (**a**) TiB_2_ and (**b**) TiAlN; (v_c_ = 800 m/min; f_z_ = 5 µm/tooth).

**Figure 17:**
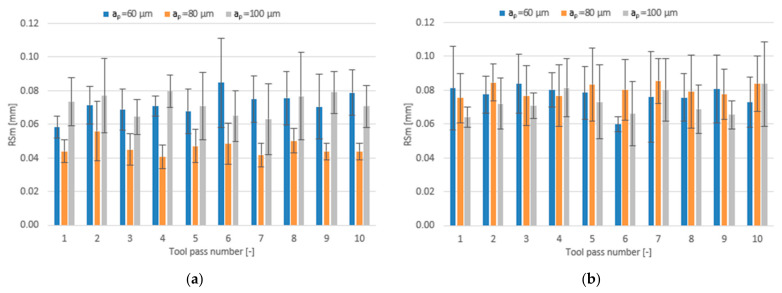
Axial depth of cut a_p_ versus surface roughness parameter RSm in the milling process performed using a tool coated with: (**a**) TiB_2_ and (**b**) TiAlN; (v_c_ = 800 m/min; f_z_ = 5 µm/tooth).

The effect of varying the axial depth of cut in the range a_p_ = 60–100 µm for different tool coating types on the surface roughness parameters is shown in the form of bar charts in Figure 15, Figure 16 and Figure 17.

The mean values of the Ra parameter obtained in the milling process conducted with the lowest and the highest axial depth of cut using the TiB_2_ -coated tool are similar, but the lowest values are obtained for the depth of cut equal to a_p_ = 80 µm. The mean values of the Ra parameter range within 0.101–0.148 µm. As for the tool with a TiAlN coating, the mean values of the Ra parameter are similar regardless of the axial depth of cut used and range within 0.119–0.159 µm.

A similar situation can be observed with respect to the surface roughness parameter Rz. In the milling process conducted using the TiB_2_-coated tool the mean values of this parameter range within 0.72–1.02 µm, while the lowest values are again obtained for the surfaces milled with the depth of cut a_p_ = 80 µm. Regarding the milling process conducted using the tool with a TiAlN coating, the mean values of the Rz parameter are similar for all tested depths of cut and range from 0.78 µm to 1.11 µm.

Like in the case of the above-discussed roughness parameters Ra and Rz, the lowest mean values of RSm not exceeding 0.056 mm are obtained in the milling process conducted with a_p_ = 80 µm using the TiB_2_-coated tool. Regarding other depths of cut, the mean values of the RSm parameter are similar and range within 0.063–0.085 mm. For the milling process performed using the TiAlN-coated tool, the mean values of this parameter are similar over the entire tested range of axial depth of cut, ranging from 0.060 mm to 0.085 mm.

**Figure 18:**
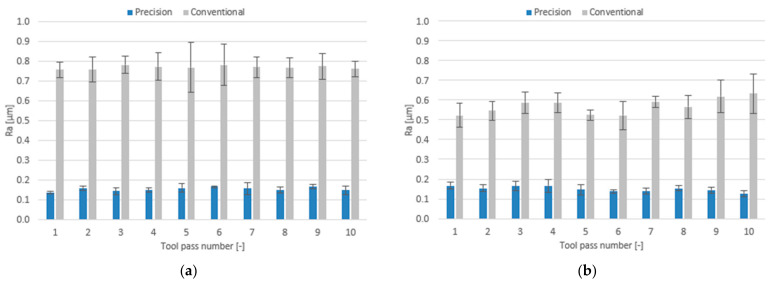
Milling conditions versus surface roughness parameter Ra in the milling process performed using a tool coated with: (**a**) TiB_2_ and (**b**) TiAlN.

**Figure 19:**
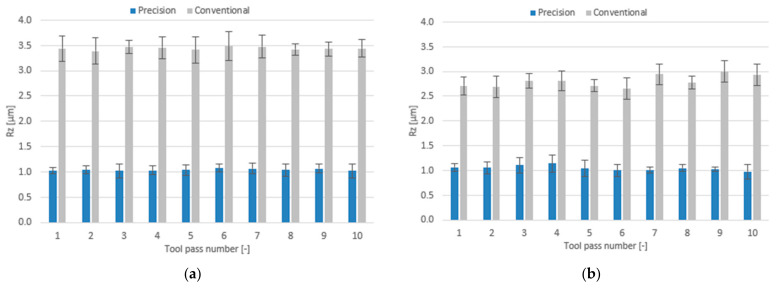
Milling conditions versus surface roughness parameter Rz in the milling process conducted using a tool coated with: (**a**) TiB_2_ and (**b**) TiAlN.

**Figure 20:**
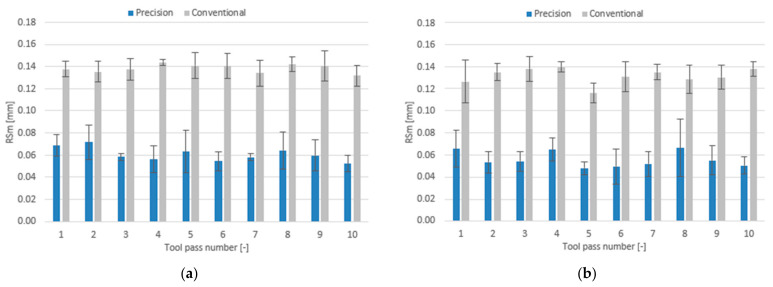
Milling conditions versus surface roughness parameter RSm in the milling process conducted using a tool coated with: (**a**) TiB_2_ and (**b**) TiAlN.

The effect of milling conditions and tool coating type on the surface roughness parameters is plotted as bar charts in Figure 18, Figure 19 and Figure 20.

For the case of precision milling, the surface roughness parameter Ra is several fold lower, regardless of the tool used. The mean values of this parameter are similar for both tools and range within 0.137–0.166 µm for the TiB_2_-coated tool and 0.127–0.166 µm for the TiAlN-coated tool. In addition, the scatter of results in the precision milling process is much lower than that obtained for conventional milling. For the case of conventional milling, the tool with a TiAlN coating turned out to be more favourable—the mean values of the Ra parameter for this tool range within 0.522–0.632 µm, while for the tool with a TiB_2_ coating the mean values of Ra range from 0.756 µm to 0.782 µm.

Similarly, much lower values of the surface roughness parameter were obtained for the surfaces subjected to precision milling. The mean values of this parameter for the tools with TiB_2_ and TiAlN coatings are similar and range within 1.02–1.07 µm and 0.98–1.14 µm, respectively. For the case of conventional milling, more favourable values of this parameter are again obtained for the TiAlN-coated tool. The mean Rz values obtained for this tool range within 2.66–3.00 µm, while for the tool with a TiB_2_ coating they range within 3.39–3.48 µm.

For the case of precision milling conditions, the surface roughness parameter RSm is several folds lower, too. The mean values of this parameter range from 0.052 mm to 0.072 mm for the TiB_2_-coated tool and from 0.048 mm to 0.066 mm for the TiAlN-coated tool. In conventional milling, the mean values of the RSm parameter are similar for both tools too, ranging 0.132–0.144 mm for the TiB_2_-coated tool and 0.116–0.140 mm for the TiAlN-coated tool.

As in the case of dimensional accuracy, the analysis of the three basic surface roughness parameters confirmed that a change in cutting speed and feed per blade had a decisive influence on the effect of the milling process. The selection of a suitable configuration of these two technological parameters makes it possible to reduce the values of the selected surface roughness parameters, while the change of the axial depth of cut again had no clear effect on the condition of the machined surfaces. Regardless of the change in the values of the technological parameters and the type of coating on the cutting tools, the obtained results were characterized by high repeatability throughout the machined surface.The results of dimensional accuracy and surface roughness were used to create Table 1 showing the percentage of the obtained dimensional tolerance range represented by surface irregularities.

The mean values of the surface roughness parameter Rz calculated for the entire surface were compared to the obtained range of dimension variability of the entire surface. The surface irregularity height obtained in milling does not exceed 40% of the dimensional tolerance range. Therefore, it can be concluded that the observed variation in dimensional accuracy not only results from surface unevenness, but it also depends on other factors. Moreover, this ratio depends on the changes in technological parameters of precision milling.

## 4. Conclusions

The experimental results of this study lead to the following conclusions:

- Obtained dimension values deviate from the assumed nominal depth along with successive tool passes.
- Increase in cutting speed causes an increased scatter of dimension values with successive tool passes and their higher deviation from the nominal depth, thus increasing the range of dimension values for the entire surface.
- Increase in feed per tooth reduces the deviation of dimension values from the nominal depth and reduces the scatter of dimension values occurring with successive tool passes, leading to a lower range of dimension variation for the entire surface.
- Change in the axial depth of cut does not have a significant impact on obtained dimension values.
- Similar dimension values are obtained for both tested tool coating types, these values being slightly more favourable for the TiB_2_-coated tool.
- Compared to conventional milling, the precision milling process makes it possible to produce surfaces with several fold lower values of the analysed surface roughness parameters and their high repeatability at individual tool passes, irrespective of the technological parameters and tool coating type used.
- Increase in cutting speed and feed per tooth leads to reduced surface roughness parameters values measured on the surfaces milled with the TiAlN-coated tool, whereas a change in the axial depth of cut does not have a direct impact on these parameters.
- For the milling performed using the TiB_2_-coated tool, the lowest values of the analysed surface roughness parameters were obtained when the milling process was conducted with intermediate values of the technological parameters.
- Similar values of the surface roughness parameters are obtained for both tested tool coating types; nevertheless, the scatter of results is smaller when the milling process is conducted with the use of the tool with a TiB_2_ coating.
- In conventional milling conditions, better surface quality is obtained when the process is performed using the tool with a TiAlN coating.
- The precision milling of magnesium alloys can be conducted with the use of conventional cutting tools and a standard CNC machine tool.

The results of this study confirm the hypothesis that, under conditions typical of machining in industrial conditions, it is possible to carry out milling process, the results of which meet the standards of precision machining and makes it possible to eliminate the need for finishing by other methods.

## Figures and Tables

**Table 1:** Ratio of surface roughness parameter Rz to dimensional tolerance range T.

$\frac{Rz}{T}[\%]$	**v_c_ [m/min]**			**f_z_ [µm/tooth]**			**a_p_ [µm]**		
	**400**	**800**	**1200**	**1**	**5**	**9**	**60**	**80**	**100**
	**TiB_2_**								
	24.0	13.6	9.9	11.6	13.6	25.4	35.5	13.6	20.0
	**TiAlN**								
	38.3	21.5	7.7	17.5	21.5	21.5	14.8	21.5	25.2

## Data Availability

The raw/processed data required to reproduce these findings cannot be shared at this time as the data also form part of an ongoing study.
